# Supplementary material for: The effect of habitual and experimental antiperspirant and deodorant product use on the armpit microbiome
Source: PeerJ. 2016 Feb 2;4:e1605. doi: 10.7717/peerj.1605 (PMC4741080; doi:10.7717/peerj.1605)

We used a 2-way ANOVA in SPSS to compare evenness by product use and sampling period. There were no significant effects of sampling time, our treatments, or their interaction on this metric of community structure.

| **Tests of Between-Subjects Effects** | | | | | |
| --- | --- | --- | --- | --- | --- |
| Dependent Variable: J' (Evenness) | | | | | |
| Source | Type III Sum of Squares | df | Mean Square | F | Sig. |
| Corrected Model | .119^a^ | 5 | .024 | 1.044 | .412 |
| Intercept | 4.759 | 1 | 4.759 | 208.070 | .000 |
| SamplingPeriod | .001 | 1 | .001 | .059 | .810 |
| ProductUse | .005 | 2 | .003 | .117 | .890 |
| SamplingPeriod * ProductUse | .112 | 2 | .056 | 2.459 | .104 |
| Error | .640 | 28 | .023 |  |  |
| Total | 5.774 | 34 |  |  |  |
| Corrected Total | .760 | 33 |  |  |  |
| a. R Squared = .157 (Adjusted R Squared = .007) | | | | | |


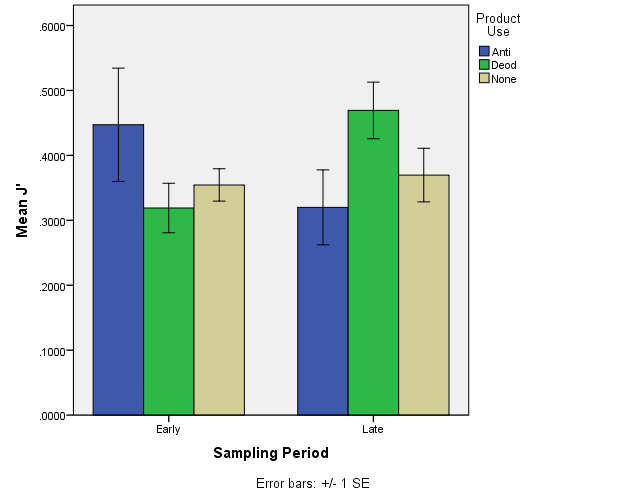

Supplement: Supplemental Information 1 — Evenness information of armpit microbes [file peerj-04-1605-s005.docx]
